# The effect of lexical triggers on Spanish-English code-switched judgment tasks

**DOI:** 10.3389/fpsyg.2024.1363935

**Published:** 2024-03-27

**Authors:** Bryan Koronkiewicz, Rodrigo Delgado

**Affiliations:** ^1^Department of Modern Languages and Classics, The University of Alabama, Tuscaloosa, AL, United States; ^2^Department of Spanish and Portuguese, University of Illinois Urbana-Champaign, Urbana, IL, United States

**Keywords:** code-switching, Spanish, acceptability judgment tasks, heritage speakers, bilingualism, methodology

## Abstract

**Introduction:**

It has been argued that certain words can “trigger” intrasentential code-switching. While some researchers suggest that cognates establish triggering at the lexical level, others have argued that words that lack direct translations are more natural stories switch. Yet to be tested experimentally is to what extent different types of lexical items influence the acceptability of mixed utterances.

**Methods:**

The current study investigates this methodological consideration for code-switching research by having early US Spanish-English bilinguals (i.e., heritage speakers of Spanish) complete an acceptability judgment task with a 7-point Likert scale directly comparing cognates (e.g., *sopa* “soup”) and culturally specific items (e.g., *pozole* “traditional Mexican soup”) in otherwise identical grammatical switched sentences (*N* = 24).

**Results:**

The results showed that there was no significant effect of condition (*p* = 0.623) suggesting that cognates and language-specific items are equally acceptable in code-switched sentences. Indeed all conditions were rated on average above 6.

**Discussion:**

These findings show that in this context, judgment tasks are not affected differently by these types of lexical items.

## 1 Introduction

Intrasentential code-switching (CS) refers to the common bilingual practice of incorporating elements from two or more languages within a single utterance. Decades of research have consistently demonstrated that this linguistic phenomenon is subject to constraints, operating according to discernible rules. That is to say, bilinguals do not mix their languages arbitrarily, but rather there are linguistic principles that govern it, mirroring the way monolingual speech is subject to grammatical, sociolinguistic, and psycholinguistic factors. By examining intrasentential CS across different linguistic contexts, scholars have been able to provide valuable insights into the systematic nature of CS and contribute to our understanding of how multilingual speakers navigate and integrate more than one language in their everyday lives. Despite general findings, the specific structural patterns regarding CS can vary from study-to-study. This variation raises methodological questions and considerations within the field of bilingualism research when it comes to the grammatical constraints on language mixing. Of course, the diversity in CS patterns may stem from factors such as the specific linguistic context being studied, including both the language pair under analysis as well as the participants' language backgrounds. However, it is also important to carefully consider the methods employed in each study. It is unclear to what extent the variability in the results is an artifact of the choices made by the researcher(s) as opposed to actual differences in linguistic structure and/or competence. As such, it is crucial to evaluate the methods employed across different studies to ensure reliable and valid conclusions. By addressing this broader methodological question of variation in CS patterns, researchers can work toward refining and enhancing their approaches, thereby contributing to a more comprehensive understanding of this complex linguistic phenomenon.

With that in mind, the present study focuses on examining different types of lexical items in an experimental Spanish-English CS task, with a specific emphasis on heritage bilingualism in the US. The primary objective is to shed light on the role different types of lexical items play in CS and determine if it influences the perceived acceptability of language mixing for this specific bilingual population. Of particular interest here are two distinct types of lexical items that seem to have been used to argue two sides of the same coin regarding the bilingual lexicon: can certain words “trigger” intrasentential CS or not? On one hand, some research suggests that a cognate (i.e., a word that has a similar or identical form and meaning across different languages) can facilitate CS at the lexical level (Broersma and De Bot, 2006; Broersma, 2009; Broersma et al., 2009; De Bot et al., 2009). This triggering hypothesis, originally developed by Clyne (as cited in Broersma and De Bot, 2006), posits that the overlap activates the bilingual speaker's other language, increasing the likelihood of a switch. Specifically, in Broersma and De Bot's (2006) adjusted triggering hypothesis, they argue that “triggering takes place at the lemma level, where the selection of a trigger word enhances the activation of the lemmas of a non-selected language” (p. 11). In other words, CS is tied to (and facilitated by) the fact that a specific lexical item has other closely related items within the bilingual lexicon. However, other research has argued that words that lack direct translations are “more natural to switch” (González-Vilbazo et al., 2013, p. 8), as some bilinguals have anecdotally reported that when there is a direct translation available, the need for a switch is nullified. From this perspective, it is when a word is language-specific that the likelihood of a switch is increased, as the bilingual speaker wants to express the specific connotations of the lexical item in its original form instead of a potentially inadequate approximation of it in their other language. Differing from the triggering hypothesis then, here the idea is that CS is tied to (and facilitated by) the fact that a lexical item *does not have* a closely related item within the bilingual lexicon.

The current investigation targets judgment data on Spanish-English CS stimuli, focusing on the potential impact of using language-specific lexical items. Consider the sentences in (1).

(1) a. He's going to serve us *sopa para la cena mañana*.
       “He's going to serve us soup for dinner tomorrow.”
    b. He's going to serve us *pozole para la cena mañana*.
       “He's going to serve us pozole for dinner tomorrow.”

Here we have two sentences that switch from English-to-Spanish. They are almost identical, except the lexical item at the switch point differs between the two, even though in both cases it is the object complement. In (1a) the switch starts at *sopa* “soup,” a direct cognate between Spanish and English. Compare this to (1b), where instead the switch begins with *pozole*, a Spanish-specific word that refers to a traditional Mexican soup that is typically made with hominy and pork. The question is whether, if all other structural components are the same, will bilingual speakers rate one type of lexical item more favorably than the other. If cognates are a stronger trigger for CS, we could expect (1a) to receive more favorable acceptability ratings due to an increase in perceived naturalness of the switch, whereas if language-specific items are the stronger trigger, we would expect the opposite where (1b) is considered more acceptable. Of course, if both are equally potent triggers, we could also expect participants to rate such sentences similarly. By taking the same exact sentences and changing the type of lexical item at the switch point, the research design allows for a systematic exploration of participants' responses and provides valuable insights into how the choice of lexical items in an experiment may shape acceptability ratings.

Overall, the findings from this study contribute to a deeper understanding of the intricate dynamics at play in CS and provide valuable methodological implications for future experimental bilingualism research. The results suggest that CS sentences with cognates and those with language-specific items receive similarly high (i.e., equally acceptable) ratings. We interpret these results to show that both types of lexical items can serve as a trigger for CS, and there is no methodological advantage to using one or the other during acceptability judgment tasks.

## 2 Background

### 2.1 Methods in code-switching research

As Gullberg et al. (2009) state in their overview of research techniques for studying CS, “the overarching methodological problem regarding experimental techniques is how to study CS without compromising the phenomenon (i.e., how to induce, manipulate, and replicate natural CS),” (p. 21). As CS research has grown more common in the field of linguistics, there have been attempts to investigate the methods being employed. Recently, for example, Jones (2023) outlines and discusses qualitative approaches to CS research. However, the current paper sits at the other end of the methodological spectrum, looking at quantitative data. In particular, it continues the conversation initiated by González-Vilbazo et al. (2013), examining the best practices in CS research that uses experimental judgment data. Since then, several different specific works have continued this thread, including studies on a wide array of issues as they relate to CS, such as stimuli modality (Koronkiewicz and Ebert, 2018), CS attitudes' effect on judgment ratings (Badiola et al., 2018), the use of control stimuli (Koronkiewicz, 2019), and the value of monolingual stimuli in CS experiments (Ebert and Koronkiewicz, 2018).

An intentionally broad overview, González-Vilbazo et al. (2013) delve into many different methodological considerations in the experimental study of grammatical aspects of CS. As they detail, the use of CS data offers a unique way to advance our understanding of linguistics, as it allows us to explore combinations of linguistic features that may remain hidden within monolingual datasets. They concentrate on three specific types of issues: (i) project design, (ii) experimental procedure, and (iii) participant selection. The current study is directly related to their first point regarding how to effectively design stimuli for a CS project. In fact, it not only relates to the topic more generally, but it directly tests one of the issues raised, which is detailed in the following section.

### 2.2 Triggering code-switching

One of the central questions at the heart of research on CS is what are the factors that contribute to the occurrence of a switch from one language to another. In De Bot et al.'s (2009) overview of the different sources of a switch, they emphasize that CS is a dynamic process influenced by a variety of internal and external factors. As they state, “there is abundant evidence for general effects of language proficiency, interactional setting, group affiliation, typological distance between languages, and various other factors that affect global patterns of [CS]” (De Bot et al., 2009, p. 85). That is to say, there is not one source of CS triggering, but rather a myriad of interrelated sources. Nevertheless, as they suggest, a better understanding of the many causes of a language switch can contribute to the development of more accurate models and theories in the field of bilingualism. The authors provide the analogy of a grain of sand that causes an avalanche; in this line of research, we are interested in identifying those grains of sand (i.e., triggers) that can provoke an avalanche (i.e., language switch).

A specific source of triggered CS that has received some attention is the lexical item. As is common in bilingual research, considerable attention has been paid to cognates, as they are a uniquely bi/multilingual lexical phenomenon. When trying to understand how and why two languages are mixed together by a speaker, it is logical to look at where the two languages overlap. There has been anecdotal support of cognates playing a role in CS since Clyne's (as cited in Broersma and De Bot, 2006) original conception of the triggering hypothesis, and it has since been supported by more recent empirical studies. In an analysis of a Dutch-Moroccan Arabic bilingual corpus, Broersma and De Bot (2006) provide statistical evidence that words that were either adjacent to or simply in the same clause as a cognate were significantly more likely to be switched. Later, Broersma (2009) found similar evidence when conducting an experiment involving Dutch-English bilingual participants. The participants completed a series of tasks that aimed to elicit CS between the two languages. The study targeted lexical activation and phonological facilitation as potential triggers, examining how they influenced language selection during speech production. The results indicated that participants were more likely to switch languages when encountering cognate words, further confirming that lexical activation plays a crucial role.

It is important to note, that although these previous studies on triggering looked at elicited CS (i.e., production), this does not mean there are disconnected from CS acceptability, which is often assessed via a receptive task. Although the triggering hypothesis centers on production data, it is still unknown how such triggering may influence a bilingual's evaluations (or judgments) after comprehension. In essence, we have yet to understand what information bilinguals use to evaluate CS sentences. It is precisely this line of reasoning that leads González-Vilbazo et al. (2013) to address lexical items as triggers for CS in their article focusing on methodological issues and considerations. However, their interest is in a sense the opposite of the previously mentioned research. Instead of looking at cognates, words that have a direct connection between the two languages, González-Vilbazo et al. point to words that seem to lack any connection whatsoever. They argue that words that lack direct translations are “more natural to switch” (González-Vilbazo et al., 2013, p. 8). They provide a Taiwanese-Spanish CS example–an item from an AJT–to illustrate their point, repeated here in (2).

(2) a. Compró Mirta *hia-e tue-chit riab ba-tzang*?
         bought Mirta those which CL ba-tzang
         “Which of those ba-tzang did Mirta buy?”
     b. Compró Mirta *hia-e tue-chit pun tse*?
         bought Mirta those which CL books
         “Which of those books did Mirta buy?”

Here we have two sentences that switch from Spanish to Taiwanese. The switch point is the same, as the entire *in-situ* wh-object is provided in Taiwanese in an otherwise Spanish question. The distinction between the two sentences is the lexical item within the wh-object, with (2a) referencing *ba-tzang*, a Taiwanese-specific word that refers to a rice dumpling, and (2b) instead referring to *tse* “books,” which has a straightforward translation. Although they did not provide experimental data, the sentence in (2b) was considered odd to their Taiwanese-Spanish consultant when trying to assess its acceptability, as she “explicitly asked why she would switch in that context, saying that switching to Taiwanese at that point seemed unnatural to her,” (González-Vilbazo et al., 2013, p. 8), leading the researchers to use (2a) in their experiment, which allowed for the assessment of the sentence to focus on the intended research question, namely the position of wh-phrases and verb-subject inversion, which differ between the two languages. In other words, had participants been provided only sentences like those in (2b), it is possible that acceptability ratings would only reflect the “unnaturalness” of the lexical item employed, instead of the syntactic underpinnings involved in the switch.

Recently, Wintner et al. (2023) also investigated lexical item triggers in CS. Conducting a corpus-based analysis of Arabic-English, German-English, and Spanish-English CS, their research aligns with the approach taken by Broersma (2009) and colleagues, as they are explicitly interested in testing the triggering hypothesis; however, they diverge in that they eschew cognates, instead focusing on what they call *shared* lexical items, which they defined as a “category of lexical items … that we expect to reside in more than one (or alternatively, in a *shared*) mental lexicon,” (Wintner et al., 2023, p. 1). In that sense, their work is more similar to González-Vilbazo et al. (2013), as they are interested in items that have no other closely related item that can be activated within the bilingual lexicon, unlike cognates. However, their definition is far more broad, as it is not limited solely to culturally specific items, as they also include proper names of individuals (e.g., *Johnson*), commercial entities (e.g., Seven *Eleven*), and geographical locations (e.g., *Times Square*), as well as international lexical items such (e.g., *taxi*) and cross-cultural social media expressions (e.g., *lol*). Overall, they found there is a strong association between CS and shared lexical items, with it potentially triggering a switch closely before or after such a lexical item's use in the bilingual corpora.

### 2.3 Research questions and hypotheses

Yet to be tested experimentally is to what extent the type of lexical item influences the acceptability of mixed utterances. As such the current study looks at lexical items in a Spanish-English CS judgment task. Although there are a number of different ways to tackle this issue, as a targeted first step we isolate our study to comparing two different types of lexical items that differ with regard to whether or not they have other closely related items in the bilingual lexicon. Specifically, we test whether the use of language-specific lexical items (e.g., *pozole* “traditional Mexican soup”) affects acceptability ratings differently than cognates (e.g., *sopa* “soup”). The explicit research question can be formulated as follows:

Research Question: Does the type of lexical item in an otherwise identical switched structure affect acceptability ratings of Spanish-English CS by US heritage speakers of Spanish?

To answer this research question, we need to simply compare the acceptability ratings of the two different conditions, (i) cognates and (ii) language-specific items. As such, there are three potential outcomes, which are articulated in the following hypotheses:

Hypothesis 1: Cognates receive higher ratings, suggesting items with other closely related items in the bilingual lexicon are perceived as more acceptable switches to participants.
Hypothesis 2: Language-specific items receive higher ratings, suggesting items without other closely related items in the bilingual lexicon are perceived as more acceptable switches to participants.
Null Hypothesis: Cognates and language-specific items will receive similar ratings, suggesting that whether an item has other closely related items in the bilingual lexicon is not a factor that participants consider when evaluating a switch.

A confirmation of Hypothesis 1 would mean that the corpus and experimental production data that found a link between cognates and CS (Broersma and De Bot, 2006; Broersma, 2009; Broersma et al., 2009; De Bot et al., 2009) also extends to bilinguals' completion of a receptive task. That is to say, it would suggest that the bilingual speakers' experience with cognates triggering CS is a more critical part of the evaluation process of judging a switched sentence's acceptability. On the other hand, if Hypothesis 2 is confirmed, it would suggest the opposite—that the bilinguals' experience with language-specific items triggering CS is more essential to evaluating such sentences. Finally, confirmation of the null hypothesis would mean one of two things: (i) it could mean that both types of experiences are involved to an equal extent when evaluating the acceptability of CS, or (ii) that neither directly matter in such a receptive task, and the bilingual participants are able to extract away from such factors and evaluate the sentences based on other characteristics (e.g., solely the syntactic structure of the switch).

## 3 Methods

### 3.1 Participants

The study focused on a group of participants who were US heritage speakers of Mexican Spanish residing in central/northern Illinois, consisting of a total of 21 individuals. The age range was from 19 to 38 years old (*M* = 29.0 years), with slightly more than half writing in their gender identity as either *female* or *woman* (*n* = 12), and the rest listing it as *male* (*n* = 8), except for one participant who chose not to disclose that information. With regard to education level, all participants, except for one, completed at least some college. A third of the participants listed having completed college as their highest education level (*n* = 6), while another third (*n* = 7) had completed a Master's degree, and one participant had completed an advanced degree (i.e., Ph.D, M.D, or J.D.).

The participants self-reported as code-switchers, indicating their tendency to mix Spanish and English during the same conversation with other bilinguals. All participants indicated that they used both languages with family members (e.g., siblings, parents, grandparents, partners, etc.), while 8 participants also included friends as bilingual conversation partners, and another 4 included people from work or school as well. Following Badiola et al. (2018), participants were asked how they felt about someone who mixes two languages in the same sentence to ensure that subjective biases about CS would not affect the results in a negative manner. The majority (*n* = 13) chose the most favorable response (*I think it's great. It shows that someone can speak well or is comfortable in both languages*.), while the next most common answer (*n* = 16) was the second most favorable response (*It's okay. It is as normal and as acceptable as speaking using the same language when talking to a person*.). The remaining participants (*n* = 2) chose the neutral response (*I do not care. I never thought about it. I do not have a strong opinion*.). Since no one chose the slightly disfavorable response (*It does not seem right. It is better to talk using the same language when I talk*) nor the completely disfavorable response (*I find it horrible. It is an aberration of the two languages. It shows that I do not speak either of them well*.), no participants were removed from the dataset due to anti-CS bias.

When asked to describe their cultural/ethnic identity, the most common answer from participants was either *Mexican(o)* or *Mexican-American* (*n* = 13). Other common answers were *Latino*/*Latinx* (*n* = 5) or *Hispanic* (*n* = 4), while one participant used *Chicano*.^1^ The vast majority of the participants were born in the US, except for 2 participants who were born in Mexico and subsequently moved to the US at an early age. Of the American-born participants, most were second-generation immigrants, as all but 2 had parents who were born outside of the US. In terms of first exposure to Spanish (*M* = 0.4 years) and English (*M* = 2.5 years), they were balanced with regard to being either simultaneous bilinguals (having been exposed to both languages before school age; *n* = 10) or early sequential bilinguals (having been exposed to Spanish at home first, and then English at school; *n* = 11).

In terms of language proficiency, the participants' Spanish skills were assessed to be at an intermediate to advanced level, with an average score of 38.3 out of 50 on a written, multiple-choice grammar and vocabulary test (Montrul and Slabakova, 2003). Their English proficiency was determined to be advanced, with an average score of 36.2 out of 40 on a parallel written, multiple-choice grammar and vocabulary test (O'Neill et al., 1981). More details about each participant's proficiency assessment scores, lexical decision task scores, as well as self-reported ratings is included in Table 1.

**Table 1:** Overview of participants' proficiency in both Spanish and English.

	**Spanish**			**English**		
**Participant**	**Vocabulary and grammar test**	**Lextale-Esp**	**Self-rating**	**Vocabulary and grammar test**	**LexTALE**	**Self-rating**
1	22	10	3.5	30	88.75	5.5
2	45	42	6	38	96.25	5.5
3	36	4	3.25	38	87.5	5.75
4	41	11	3	35	91.25	5
5	37	24	3.25	37	98.75	5.5
6	47	47	5.25	37	100	5.75
7	43	12	4.75	36	75	6
8	37	44	4	36	91.25	6
9	37	17	3.25	37	96.25	5.25
10	45	41	4.5	39	98.75	6
11	37	0	5.25	34	80	6
12	32	−1	5.25	37	86.25	6
13	41	12	5	35	75	6
14	22	12	3	39	75	6
15	45	35	5	37	83.75	5
16	35	12	5.25	38	75	6
17	31	5	5	36	93.75	6
18	47	52	5.75	37	96.25	5.25
19	38	32	4.5	31	77.5	5.25
20	45	17	3.5	37	96	5.25
21	41	12	5.25	37	75	6

The subjective self-ratings are an average score taken from the four questions of the Bilingual Language Profile (Birdsong et al., 2012) related to their own assessment of their speaking, understanding, reading, and writing skills in both languages on a scale from 0 to 6. The vocabulary and grammar tests both consisted of a series of multiple-choice questions, with a maximum score of 50 for Spanish (Montrul and Slabakova, 2003) and a maximum score of 40 for English (O'Neill et al., 1981). Finally, the two lexical decisions tasks followed the scoring procedure outlined by the respective authors, with the Lextale-Esp (Izura et al., 2014) being scored from −60 to 60, and the LexTALE from 0 to 100 (Lemhöfer and Broersma, 2012).

### 3.2 Procedure

The study was completed via the online questionnaire software Qualtrics. There were two different surveys that participants completed on separate days. First, after filling out a consent form, participants completed two language-specific lexical decisions tasks, first the English LexTALE (Lemhöfer and Broersma, 2012) and then the Spanish Lextale-Esp (Izura et al., 2014). Afterwards, participants filled out some basic background questions about their language history with English and Spanish, as well as their use and attitudes toward CS. On average, this initial survey took about 15 min to complete.

Once a participant completed the first survey, they were provided a link to a second one. After filling out an additional consent form, participants completed a brief training session on the experimental task, which was an acceptability judgment task (AJT). An AJT taps into a person's linguistic intuitions, asking them to use their innate sense of what sounds natural or grammatically correct in their language(s). By collecting acceptability judgments, linguists can access this implicit knowledge and gain insights into the underlying rules and constraints of a language, or in this case language mixing. The first goal of the task training was to provide an overview of what is meant by linguistic acceptability, explaining that we were interested in “the linguistic structures that you have in your mind as a bilingual speaker,” not prescriptive rules or just general comprehensibility of the sentences. The second goal follows González-Vilbazo et al.'s (2013) methodological recommendation to prime the participants into CS mode by providing the entire training sequence in a mixture of Spanish and English. For example, the training starts with the following sentence: “In this study we are interested in finding out *cómo funciona el code-switching de inglés a español y vice-versa*” (“... how code-switching from English to Spanish works and vice-versa”). Not only did this prime CS mode, by including written CS in the study materials, we aimed to alleviate any potential formality bias against language mixing, as participants could intuit that the research being conducted considered CS to be a completely acceptable way to use the two languages.

**Figure 1:**
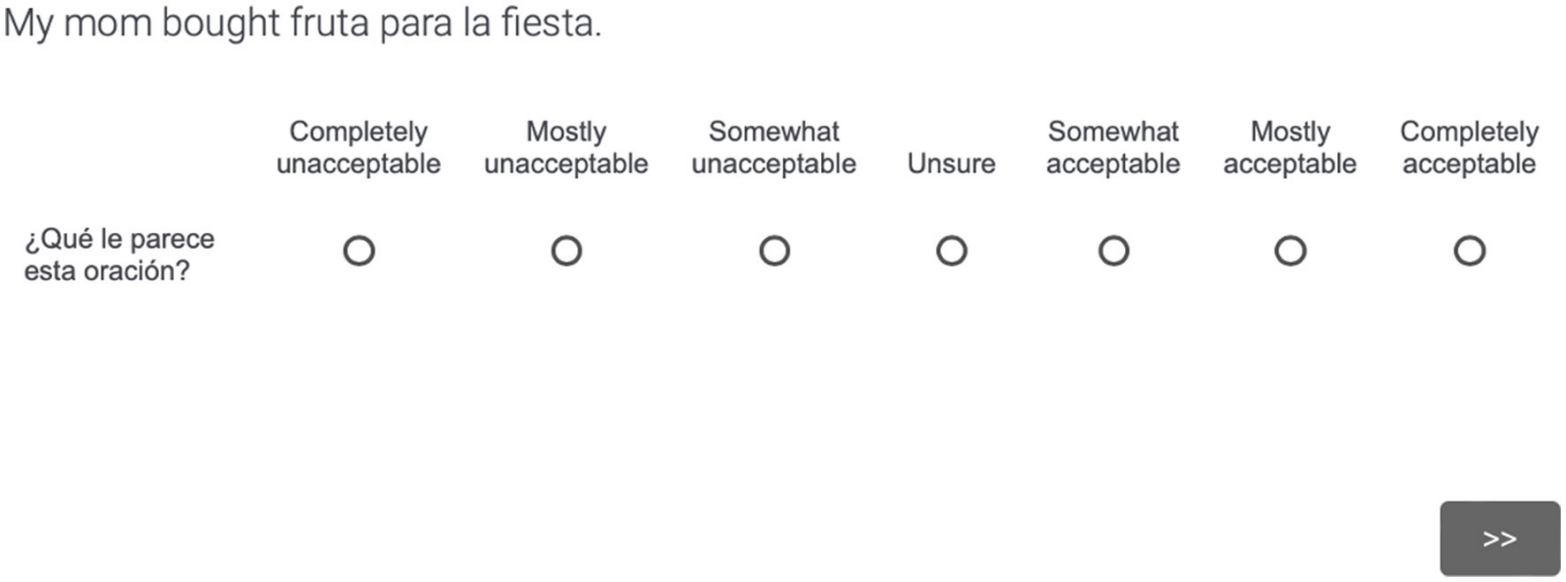
Sample CS AJT item.

After the training, participants completed two distinct randomized blocks of CS stimuli. The blocks were separated by half of the background questionnaire; this first portion included the first two sections of the Bilingual Language Profile (Birdsong et al., 2012), which focused on language history and language use. Within both AJT blocks, each stimulus was presented one at a time with a 7-point Likert scale. An example of an AJT item is provided in Figure 1. As shown, the participants were prompted with the question ¿*Qué le parece esta oración?* “How does this sentence seem to you?” and they were provided with the following labels on the Likert scale: 1 = *Completely unacceptable*, 2 = *Mostly unacceptable*, 3 = *Somewhat unacceptable*, 4 = *Unsure*, 5 = *Somewhat acceptable*, 6 = *Mostly acceptable*, and 7 = *Completely acceptable*. The target stimuli for the current study were in the second block of CS stimuli, whereas the first block investigated a different methods-based project unrelated to lexical-item choice.

After completing all of the CS judgments, participants were given the Spanish proficiency measure^2^ (Montrul and Slabakova, 2003), which consisted of both a series of sentence-level multiple-choice vocabulary questions as well as a multi-paragraph passage that targeted both vocabulary and grammar via multiple-choice blanks. The Spanish proficiency measure was followed by a block of stimuli that were entirely in Spanish with the same set up as the CS blocks, with the only difference being that the Likert scale for the AJT was changed to Spanish labels: 1 = *Completamente inaceptable*, 2 = *Mayormente inaceptable*, 3 = *Un poco inaceptable*, 4 = *No sé*, 5 = *Un poco aceptable*, 6 = *Mayormente aceptable*, and 7 = *Completamente aceptable*. After the Spanish block, the participants then completed the English proficiency measure (O'Neill et al., 1981), which was a similar multi-paragraph passage with multiple-choice blanks, followed by a block of English-only stimuli. Here the original English labels were used again for the Likert scale, while the prompt question for the AJT was changed to English, asking *How acceptable is this sentence?* Finally, the participants completed the second half of the background questionnaire, which included the third and fourth portions of the Bilingual Language Profile (Birdsong et al., 2012), targeting language proficiency and language attitudes. In general, this second longer survey took about 75 min to complete. For completing both parts of the study participants were compensated $25 via Amazon e-codes.

### 3.3 Stimuli

The target CS stimuli included written sentences (*N* = 24) with three different types of object complement switches: adjectives (*N* = 8), as in (3); transitive direct objects (*N* = 8), as in (4); and ditransitive direct objects (*N* = 8), as in (5).

(3) a. He was called *estúpido/naco todo el tiempo*.
         “He was called stupid/naco all the time.”
      b. *Le llamaba* stupid/boneheaded all the time.
         “They called him stupid/boneheaded all the time.”
(4) a. My mom bought *fruta/nopales para la fiesta*.
         “My mom bought fruit/nopales for the party.”
      b. *Mi mamá compró* fruit/crackers for the party.
         “My mom bought fruit/crackers for the party.”
(5) a. He's going to serve us *sopa/pozole para la cena mañana*.
         “He's going to serve us soup/pozole for dinner tomorrow.”
      b. *Nos va a servir* clam chowder/soup for dinner tomorrow.
         “He's going to serve us clam chowder/soup for dinner tomorrow.”

Regarding switch direction, for these complement structures the syntactic frames are comparable between the two languages (Zagona, 2012). Moreover, object complement switches are commonly reported throughout the CS literature, both in experimental contexts and in naturalistic data (Poplack, 1980; Sankoff and Poplack, 1981; MacSwan, 1999; Toribio, 2001; Muysken, 2007; among others), and they have been used as a default acceptable switch site in previous research as well (Anderson and Toribio, 2007); as such all of the sentences in (3–5) are expected to be completely acceptable for heritage speakers of Spanish in the US, at least from the standpoint of the syntactic structure.

Within each type, there were two pairs of a semantically parallel cognate and language-specific items in Spanish, and another two pairs in English. That is to say, for each type of object switch, there were 4 stimuli that switched from English-to-Spanish at the target item—as in (3a), (4a), and (5a)—and 4 stimuli that switched from Spanish-to-English—as in (3b), (4b), and (5b). As shown, the only difference between the two conditions was the target item, and as such, any difference in acceptability would be due to the lexical item choice and not any other factor. To create the two groups, we started with the language-specific items in Mexican Spanish, a list of which was created where there were no other closely related English lexical items in the bilingual lexicon. These items included: *naco, chicano, nopales, champurrado, atole*, and *pozole*. Sentence frames were then created around these items. Next, English lexical items were compiled that would fit those same frames but also had no other closely related Mexican Spanish word for them. These included: *boneheaded, snooty, crackers, seltzer, biscuits*, and *clam chowder*. Finally, cognates between the two languages (i.e., words that overlapped in terms of pronunciation and spelling) were selected that would fit those same frames. The cognates used were: *stupid/estúpido, native/nativo, fruit/fruta, coffee/café, pancakes/panqueques*, and *soup/sopa*.

The target stimuli were randomized alongside filler stimuli (*N* = 54). These sentences focused on preposition stranding (6), pied piping (7),^3^ and switch direction (8). Importantly, the filler items as well as the items from the first block of CS judgments (9) were expected to receive a mixture of acceptable and unacceptable ratings from participants, thus ensuring their use of the full scale through the sequence of AJTs.

(6) a. ¿*Qué hombre* is Ashley dancing with?
         “What guy is Ashley dancing with?”
     b. ^*^ ¿What guy está *bailando Araceli con*?
         “What guy is Araceli dancing with?”
(7) a. ¿*Con qué hombre* is Ashley dancing?
         “With what guy is Ashley dancing?”
     b. ^*^ ¿With what guy *está bailando Araceli*?
         “With what guy is Araceli dancing?”
  (8) a. *Yo fui a la fiesta* with my friend.
         “I went to the party with my friend.”
     b. I went to the party *con mi amigo*.
         “I went to the party with my friend.”
(9) a. *Los estudiantes* have paid attention to the professor today.
         “The students have paid attention to the professor today.”
     b. ^*^ *Los estudiantes han* paid attention to the professor today.
         “The students have paid attention to the professor today.”
     c. *Hace un minuto yo pedí* a beer at the bar.
         “A minute ago I ordered a beer at the bar.”
     d. ^*^ *Hace un minuto yo* ordered a beer at the bar.
         “A minute ago I ordered a beer at the bar.”
     c. *La biblioteca no abre* on Sunday mornings.
         “The library does not open on Sunday mornings.”
     d. ^*^ *La biblioteca no* open on Sunday mornings.
         “The library does not open on Sunday mornings.”

As argued by Ebert and Koronkiewicz (2018), to be sure that the judgments provided by participants are tied directly to the switch and no other aspect of the sentence, a parallel set of stimuli were also tested that were monolingual. These items were the exact same as the ones presented in the CS blocks but entirely in one language or the other. For example, while participants first rated Spanish-to-English sentences, like in (3a), and the complementary English-to-Spanish versions, like in (3b), in the CS AJT blocks, they also then rated the Spanish-only versions of those sentences, as in (10), and the English-only versions, as in (11), in separate subsequent blocks.

(10) Le llamaba estúpido/naco todo el tiempo.
         “They called him stupid/boneheaded all the time.”
  (11) He was called stupid/boneheaded all the time.

### 3.4 Analysis

Once the results were collected from all the participants, they were pre-processed to ensure the reliability and validity of the dataset. First, there were no missing or incomplete responses, and as such no trials were excluded. Next, due to the potential for individual participants to employ diverse and idiosyncratic interpretations of Likert scale values, we standardized the judgment ratings into *z*-scores, adhering to the pre-processing recommendations outlined by Schüte and Sprouse (2014). This particular computation serves to enhance the reliability of comparisons across participants by assisting in the mitigation of potential scale bias. To do so, first a mean judgment rating was calculated for each participant (across the whole experiment, not just the target stimuli for this study), and the same was done regarding the standard deviation of those ratings. With that information, each individual Likert-scale rating was converted to a *z*-score using the formula *z* = (x-μ)/σ, where x is the raw Likert score (from 1 to 7), μ is the mean judgment rating for that particular participant, and σ is the standard deviation of the ratings for that same participant.

Another important step in the analysis of the current study's data is to ensure that the task was effective at eliciting acceptability judgments. Recall that the stimuli under analysis here are all expected to be grammatical, so with that information alone it would be difficult to be sure that the task was able to elicit the participants' acceptability. How can we know if the AJT is capable of showing differences in acceptability ratings if the target stimuli are expected to be rated the same? Although the first CS block was included in the overall procedure for a separate project, we can use the data from it as a measure of task effectiveness, as it explicitly included pairs of grammatical and ungrammatical switches targeting pronouns, auxiliary verbs, and negation, as exemplified above in (9). A Welch's two sample *t*-test was conducted to compare the *z*-score ratings for these switches, and indeed the grammatical switches received significantly higher mean ratings (0.21) compared to the ungrammatical switches (−1.58), *t*_(444.78)_ = 22.538, *p* < 0.001, suggesting that the AJT was able to tap into such differences in acceptability.

## 4 Results

Returning to the current research question, we can begin our presentation of the results by reporting the Likert scale ratings that were provided by the participants, comparing the two stimuli conditions across the four different language conditions. These are presented in Table 2. First and foremost, we can see that, as expected, participants rated all of these sentences on the higher end of the acceptability scale, as all of the mean ratings are well above 4, which was the middle point of the scale provided. As we can also see, the mean ratings for the two conditions are extremely parallel.

**Table 2:** Overall raw Likert ratings for each language(s) by condition.

	**Cognate**		**Language-specific**	
	***M***	***SD***	***M***	***SD***
English	6.89	0.48	6.87	0.68
Spanish	6.53	1.34	6.47	1.51
Spanish-to-English	6.03	1.93	6.32	1.56
English-to-Spanish	6.41	1.43	6.58	1.20

**Figure 2:**
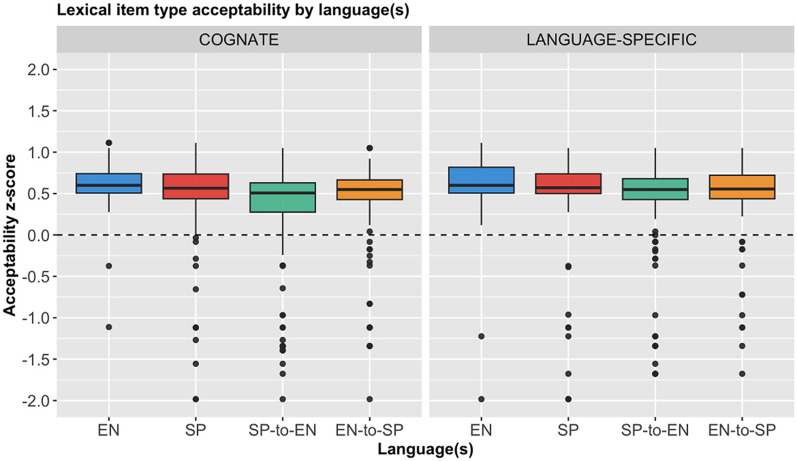
Overall z-scores for each language(s) by condition.

As the uppermost end of the Likert scale was designated as the most acceptable, when interpreting the *z*-score conversion, a sentence type's perceived acceptability by participants, relative to their overall mean rating, is directly reflected by the positivity of the mean *z*-score. Conversely, a sentence type is considered more unacceptable when the mean *z*-score is more negative. These standardized ratings are presented in Figure 2.

As we can see, the overall picture remains the same, as the sentences were rated as generally acceptable by the participants. Most importantly, though, we can see clearly that the results for both the cognates and the language-specific items are indeed identical.

A repeated measures ANOVA with participant and stimulus item as within-subject variables was chosen as the parametric statistical because it allows us to compare lexical items' influence on acceptability ratings within switched structures, while controlling for individual differences among participants and potential variability in stimulus items. There was a significant main effect of language on the *z*-score ratings, *F*_(3, 952)_ = 2.930, *p* = 0.033. However, there was no significant main effect of condition, *F*_(1, 952)_ = 0.243, *p* = 0.623, nor was there a significant interaction between condition and language *F*_(3, 952)_ = 0.290, *p* = 0.832. *Post-hoc* analysis using pairwise *t*-test comparisons with a Tukey correction showed that the English sentences received significantly higher ratings than all the other language conditions (*p* < 0.01), while the Spanish-to-English sentences received significantly lower ratings than all the others (*p* < 0.01; meaning the Spanish and English-to-Spanish sentences occupied a sort of middle ground with regard to overall acceptability).

**Figure 3:**
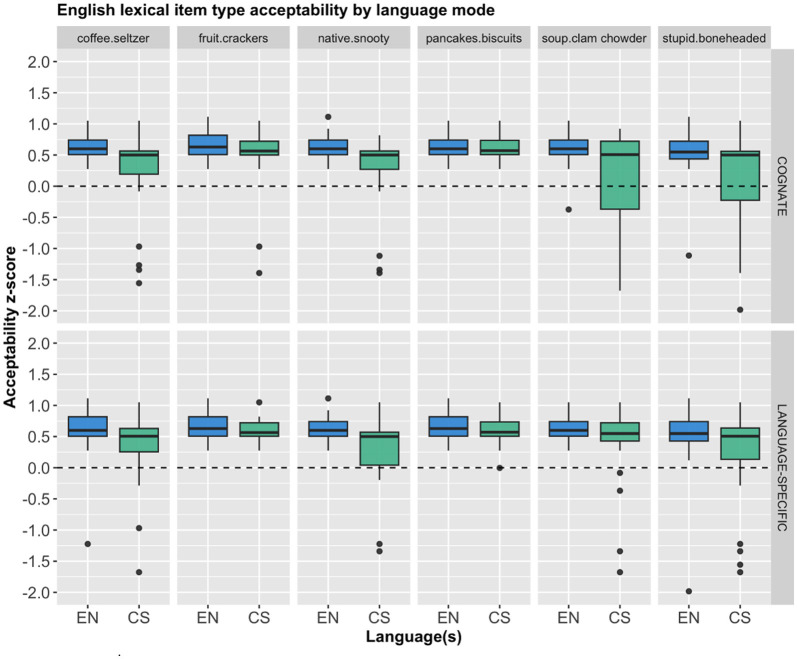
English lexical item acceptability for each language mode by condition.

**Figure 4:**
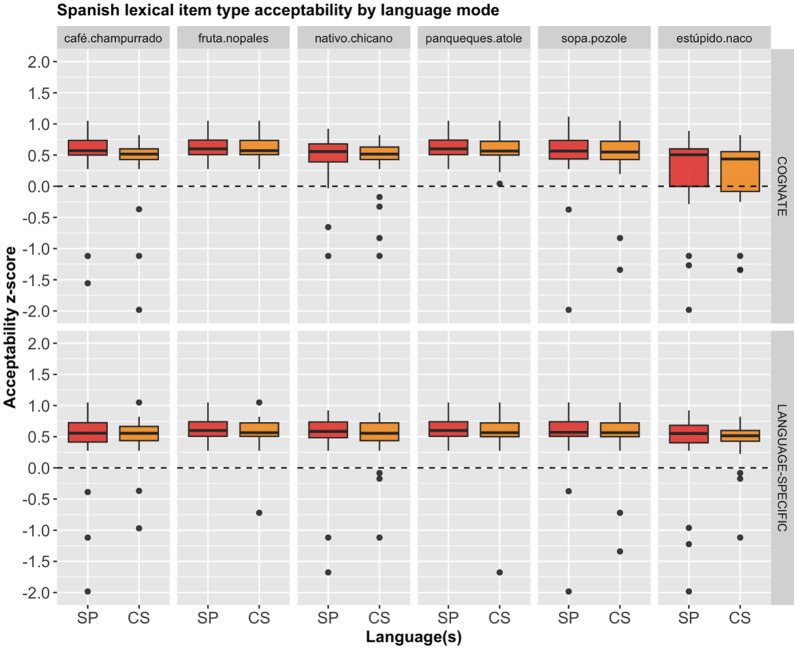
Spanish lexical item acceptability for each language mode by condition.

Although the overall pattern with regard to the two conditions seems to be rather straightforward, it is worth looking more closely at the individual lexical items. Therefore, we provide the mean *z*-score acceptability of each lexical item for both English and Spanish, as shown in Figures 3, 4, respectively. Descriptively, there seems to be minimal variation based on the individual lexical items, as the same pattern holds, where the cognates and the language specific items received almost identical acceptable ratings across the board.

A separate repeated measures ANOVA with participant and stimulus item as within-subject variables showed that there was no significant main effects on the English lexical items' *z*-score ratings for: item type, *F*_(5, 468)_ = 1.285, *p* = 0.269; condition, *F*_(1, 468)_ = 0.513, *p* = 0.474; nor an interaction between the two, *F*_(5, 468)_ = 1.313, *p* = 0.257. Similarly, a different repeated measures ANOVA with participant and stimulus item as within-subject variables showed that there was no significant main effects on the Spanish lexical items' *z*-score ratings for: item type, *F*_(5, 468)_ = 2.163, *p* = 0.057; condition, *F*_(1, 468)_ = 0.000, *p* = 0.999; nor an interaction between the two, *F*_(5, 468)_ = 0.120, *p* = 0.988.

## 5 Discussion

Our results support the null hypothesis, indicating that the types of lexical items included here do not affect judgments in AJTs differently. Both sides of the “debate” appear valid—cognates and language-specific items demonstrate a positive effect, as evidenced by their high ratings. This finding is a first step toward easing the researcher's burden when selecting lexical items for an AJT; our data suggests that whether or not a word has other closely related items in the bilingual lexicon should not impact judgments. Researchers can, therefore, include either item type and focus on testing the structure without undue concern about which type was included.

It may seem improbable that the two conditions that were tested would have similar effects. With language-specific words, it is easier to understand why CS is triggered—it is challenging to think of the English version of *pozole*, especially in the middle of an utterance. As such, when presented with such a scenario, bilinguals are able to embrace the full use of their linguistic repertoire by using CS. Although such examples for many bilinguals are completely unrelated to proficiency, such switching is in line with language mixing that arises from lexical gaps. However, with cognates, we can still ask the following question: Why switch? Why is *sopa* equally acceptable as *pozole* when they could just have easily used English entirely? In this case, it might not be a matter of the bilingual lexicon but rather the speaker's choice. As Bullock and Toribio (2009) suggest, “for many bilinguals, CS merely represents another way of speaking; that is, some bilinguals code-switch simply because they can” (p. 11). Then, can we consider this as “triggering” CS? We cannot say for certain, as our data only taps into acceptability. Nevertheless, we have demonstrated that cognates and language-specific items are not likely confounding variables in AJTs. This conclusion then applies specifically to experiments where the purpose is not necessarily to trigger CS, such as examining p-stranding, adverb order, and so on in mixed sentences. It might play a bigger role, however, in experiments where forcing a trigger is necessary.

Several results were surprising, one being that English monolingual sentences received slightly higher ratings. One possible explanation is that these participants are English dominant, as indicated by their scores on the Bilingual Language Profile (Birdsong et al., 2012). However, although significant, the difference in averages between English monolingual sentences (highest average) and Spanish-to-English (lowest rating) was only 0.89 (*p* > 0.001), < 1 point on the 7-point scale; that is to say, they were not rated on opposite ends of the acceptability spectrum. Another surprising result was that Spanish-to-English was the lowest-rated condition, contrary to expectations based on previous research, which has shown that the direction of the switch tends be toward the language with superior status or the language of power (Blokzijl et al., 2017). Nevertheless, all conditions were rated above 6 out of 7 on average, leading to the safe conclusion that, in the context of AJTs, these two lexical item types (i.e., cognate vs. language-specific) and language direction play a minor role in acceptability.

Our study has limitations, prompting avenues for future research. These results cannot be generalized to all code-switchers in the US, as we assume, following De Bot et al. (2009), a distinction between occasional and habitual CS. Researchers can explore if there is a difference between these two populations. For example, a future study could employ the Bilingual Code-Switching Profile (Olson, 2022) to see whether variations in speakers' reported experience and engagement with CS show different results with regard to lexical items in AJTs. Missing in this study are lexical items that are neither cognates nor language-specific (e.g., *food/comida*). These should be included to examine a middle ground with regard to related items in the bilingual lexicon. The conclusions that we make in this paper only apply to the two conditions tested. Along the same vein, number words could be a fruitful avenue, as it has been shown “that both backward and forward translation of number words yields a semantic number magnitude effect… providing evidence for strong form-to-meaning mappings” (Duyck and Brysbaert, 2008). In other words, it takes more time to translate number words that represent large quantities than smaller quantities. It would be interesting to see if this dichotomy triggers CS. Finally, in this experiment we only included three different types of object complement switches: (i) adjectives, (ii) transitive direct objects, and (iii) ditransitive direct objects. Future studies should explore other switch types and different lexical items.

## 6 Conclusion

In conclusion, our study contributes valuable insights to the understanding of CS and its relationship with whether or not is triggered by the (un)availability of related items in the bilingual lexicon. All conditions received high ratings, affirming that, in the context of AJTs, lexical item type (i.e., cognate vs. language-specific) and language direction play a minor role in acceptability. Importantly, this finding alleviates concerns for researchers selecting lexical items for AJTs, indicating that including cognates instead of lexically-specific items or vice versa does not significantly impact judgments. Significantly, this paper marks an initial step in laying the groundwork in choosing the proper lexical items for the design of experimental CS projects.

## Data availability statement

The datasets presented in this study can be found in online repositories. The names of the repository/repositories and accession number(s) can be found at: https://github.com/rdelga21/lexical_triggers.

## Ethics statement

The studies involving humans were approved by the University of Illinois at Urbana-Champaign, Office for the Protection of Research Subjects, IRB #23376. The studies were conducted in accordance with the local legislation and institutional requirements. The participants provided their written informed consent to participate in this study.

## Author contributions

BK: Conceptualization, Data curation, Formal analysis, Funding acquisition, Investigation, Methodology, Project administration, Resources, Visualization, Writing – original draft, Writing – review & editing. RD: Conceptualization, Data curation, Formal analysis, Funding acquisition, Investigation, Methodology, Project administration, Resources, Visualization, Writing – original draft, Writing – review & editing.

## Footnotes

^1^The different terms listed here do not add up to the number of participants because some used a combination of two, such as *Mexican/Hispanic* or *Latino/Mexicano*.

^2^This proficiency measure is often (somewhat inaccurately) referred to as the modified DELE (*Diplomas de Español como Lengua Extranjera*) due to it originally being adapted from a portion of internationally recognized certifications that assess proficiency in the Spanish language by the Instituto Cervantes; however, although this version is still regularly used in linguistic research, it no longer reflects the current DELE.

^3^The sentences in (6) and (7) are part of a separate project (Delgado and Koronkiewicz, in preparation), and were used as fillers for the current project. In the separate study, Spanish-English bilinguals, dispreferred *p*-stranding for English-to-Spanish (6a) (M = −1.00, SD = 1.03) compared to pied-piping (M = −0.32, SD = 0.97), while for Spanish-to-English it was the inverse, as *p*-stranding was more acceptable CS (M = −0.03, SD = 0.85) than pied-piping (7b) (M = −0.55, SD = 1.06). For this reason, (6a) and (7a) as marked as acceptable, and (6b) and (7b) are marked as unacceptable.
